# The Advancement of the Electrochromic Supercapacitor Properties of Interface-Engineered Hybrid Polyaniline/Prussian Blue Thin-Film Electrodes

**DOI:** 10.3390/polym18050583

**Published:** 2026-02-27

**Authors:** Suhas H. Sutar, Vinayak S. Jadhav, Dhanaji S. Dalavi, Supriya A. Patil, Sejoon Lee, Sangeun Cho, Deepak R. Patil, Nabeen K. Shrestha, Sarfraj H. Mujawar, Akbar I. Inamdar

**Affiliations:** 1 Department of Physics, Yashavantrao Chavan Institute of Science Satara, Karmaveer Bhaurao Patil University, Satara 415001, India; 2 Department of Applied Science and Engineering, Dnyanshree Institute of Engineering and Technology, Satara 415013, India; 3 Department of Physics, Krishna Mahavidyalaya, Rethare Bk, Satara 415108, India; 4 Department of Nanotechnology and Advanced Materials Engineering, Sejong University, Seoul 05006, Republic of Korea; 5 Division of System Semiconductor, Dongguk University, Seoul 04620, Republic of Korea; 6 Department of Physics, Dongguk University, Seoul 04620, Republic of Korea

**Keywords:** energy storage, supercapacitor, electrochromism, conducting polymers, thin film

## Abstract

There is an increasing demand for multifunctional devices, that can operate simultaneously as energy storage and electrochromic display devices, widely known as electrochromic supercapacitors. For instance, Prussian blue (PB) exhibits outstanding electrochromic properties; however, it has not been well explored for energy storage applications. Moreover, the electrochemical properties can be enhanced by surface engineering the host material via compositing with conducting polymers. In this work, we studied the electrochromic supercapacitor properties of composites such as Prussian blue-polyaniline (PB-PANI). The PB-PANI 90 composite thin-film electrode exhibited the highest coloration efficiency of 461.39 cm^2^/C and demonstrated superior electrochemical performance, with an aerial capacitance of 50.80 mF/cm^2^ and an optical modulation of 19.4%. All samples achieved rapid switching times of less than 3 s. These findings highlight the potential of optimizing conducting polymer coatings on Prussian blue to achieve a well-balanced composite structure with enhanced morphological properties, paving the way for advanced multifunctional electrochromic supercapacitor devices in next-generation smart systems.

## 1. Introduction

Conducting polymers were discovered by pioneering scientists Shirakawa, Heeger, and MacDiarmid in the 1970s [1,2]. Since then, conducting polymers have attracted significant interest due to their vast potential for various electronic devices, such as electrochromic displays (ECDs), light-emitting diodes (LEDs), field-effect transistors (FETs), organic-based solar cells, and chemical sensors and supercapacitors [3,4]. Their unique electrical and optical properties make them highly suitable for these applications. Moreover, the ease of the polymer synthesis process, including faster reaction rates, is one of the key factors encouraging scientists to invest effort in these domains [5,6,7]. Polymers are outstanding candidates for electrochromic display devices. The electrochromic process refers to the ability of a material to show optical changes due to surface reactions in an electrolyte containing redox-active ions. Additionally, these materials can store energy as a supercapacitor via the redox reaction. Thus, the combination of electrochromic and supercapacitor behaviors of materials is known as an electrochromic supercapacitor, due to their similar working principles [8,9]. Ongoing advancements in electrochromic energy storage devices have searched extensively for materials with multi-color switching abilities. Conducting polymers are gaining interest for such applications due to their contribution to energy storage, along with a variety of colors in different redox active states [10,11,12]. To overcome the limitations of a single metal oxide or polymers, the fabrication of composite or hybrid materials has become a crucial strategy to enhance the electrochromic and storage behaviors of the materials [13,14]. Therefore, a composite of polymers like polyaniline (PANI) with Prussian blue is being explored, considering Prussian blue is an inorganic material and an effective candidate for energy storage applications [15].

As we all know, Prussian blue is the best candidate for energy storage applications and smart electrochromic displays; the performance is further enhanced by doping with different elements such as conducting polymers. This doping enhances the overall electrical conductivity of the electrode material, leads to morphological advancement, and acts as a composite/hybrid material (transition metal oxides and conducting polymers) for energy storage applications [14,16]. One of the disadvantages of Prussian blue is that it is unstable during electrochemical studies in alkaline and acidic electrolytes. Also, the energy storage performance of Prussian blue is exceptionally low as far as dual functionality is concerned. Therefore, the strategy to deposit polymers on the electrodeposited Prussian blue films is used to assess the sustainability and electrochromic effect of the supporting layer along with the color responsiveness. The complex structure of PB does not support more structural modifications, so layer-by-layer deposition is mostly used by researchers. Moreover, Prussian blue analogues (PBAs) are also explored by researchers for different applications, replacing Fe (II) in the structure with different suitable elements [17,18,19]. Based on the literature survey, conducting polymers like PANI are widely prepared via electrodeposition, which makes it a suitable composite material with Prussian blue. The combined effects can be verified with suitable electrolytes as targeted polymers show electrochromic effects in H_2_SO_4_ while PB in KCl.

In this study, Prussian blue films are deposited onto indium-doped tin oxide (ITO)-coated glass substrates by using the chronopotentiometric technique with a constant current pulse, whereas PANI films are grown at constant potential pulses simultaneously. Electrochemical and optical studies have been conducted to investigate the effect of the combined contributions of PB/PANI films. Moreover, the optimization of reaction times to obtain the highest electrochromic supercapacitor performance is assessed. The PB-PANI 90 composite thin-film electrode exhibited enhanced electrochemical properties in terms of coloration efficiency, aerial capacitance and optical modulation.

## 2. Experimental Details

### 2.1. Materials

Potassium ferricyanide (K_3_Fe(CN)_6_), Ferric chloride (FeCl_3_), Aniline, Pyrrole, Sulphuric Acid (H_2_SO_4_) and Potassium chloride (KCl) were purchased from Loba Chemie Pvt. Ltd., Mumbai, India. The conducting substrates of In:SnO_2_ (ITO) with sheet resistance <6 ohm/sq with optimal transmittance up to 83% and thickness 1.1 mm were purchased from Vritra Technologies, Delhi (India).

### 2.2. Synthesis of Prussian Blue (PB) Films

Prussian blue thin films were synthesized on ITO substrates using chronopotentiometry technique. Prior to that, the ITO substrates were cleaned using ultrasonic treatment in dilute acid, ethanol, and double-distilled water for 5 minutes each. Thin films were deposited using a three-electrode electrochemical system connected to a Potentiostat (Metrohm M205, Herisau, Switzerland), where the pre-cleaned ITO substrate served as the working electrode, a platinum wire acted as the counter electrode, and an Ag/AgCl electrode was used as the reference electrode. The solution in the reaction bath consisted of 0.01 M of K_3_Fe(CN)_6_, 0.01 M of FeCl_3_, and 0.1 M of KCl in 100 mL of double-distilled water. Further Prussian blue films were deposited at an applied current pulse of 50 μA/cm^2^ for four hundred seconds. The resulting blue-colored films were air-dried at 50 °C for a specific period before being subjected to the electrodeposition of polymers.

### 2.3. Electrodepotion of Polyaniline (PANI)

The electrodeposition of polyaniline was conducted via the chronoamperometric technique, where a constant voltage pulse was applied for varying times. The electro-polymerization of PANI was performed at 0.9 V in a solution bath containing 0.1 M of aniline monomer and 0.5 M of H_2_SO_4_ in 100 mL of double-distilled water. Three different films were prepared on PB at varying reaction times of 30, 60, and 90 s. These films were denoted as PB-PANI 30, PB-PANI 60 and PB-PANI 90, respectively. Finally, all the deposited films were rinsed multiple times in DI water and dried in air.

### 2.4. Physicochemical and Electrochemical Property Measurements

The presence of pure-phase PB films without any impurities was confirmed using X-ray diffraction (XRD) analysis, conducted with a Rigaku MiniFlex 600 (Rigaku Corporation, Tokyo, Japan) diffractometer utilizing Cu Kα radiation (wavelength: 0.154 nm). The surface morphology at various magnifications and elemental compositions was examined through scanning electron microscopy (SEM) using a JEOL JSM-IT200 (JEOL Ltd. Akishima, Japan) system. Optical properties and potential variations were assessed through transmittance measurements using a Shimadzu UV-2600i UV-VIS spectrophotometer (Shimadzu Corporation, Kyoto, Japan). Electrochemical performance, including cyclic voltammetry, galvanostatic charge–discharge, electrochemical impedance spectroscopy, and chrono-based methods, was evaluated using a three-electrode setup operated through a Metrohm Multi Autolab M204 (Metrohm Autolab, Utrecht, The Netherlands) electrochemical workstation.

## 3. Results and Discussion

**Figure 1:**
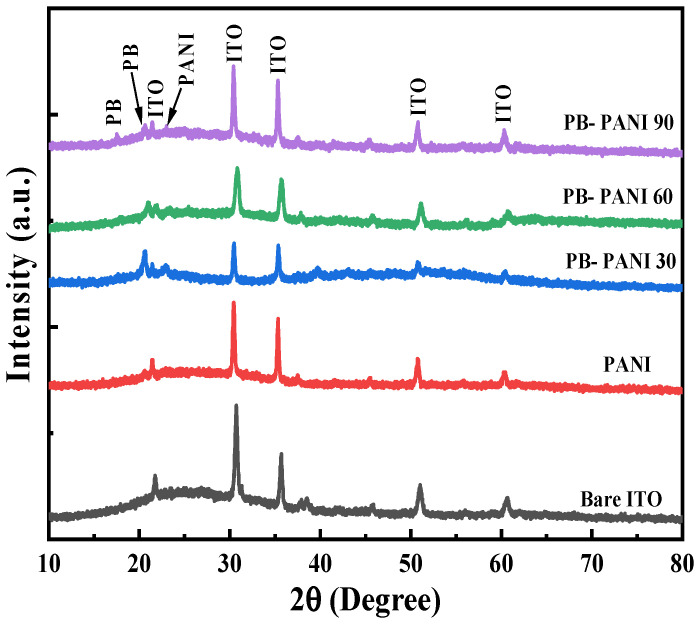
X-ray diffraction patterns of composite PB-PANI thin-film electrodes fabricated at different times, along with the pristine PANI and bare ITO.

The X-ray diffraction (XRD) patterns of all samples prepared at different reaction times are presented in Figure 1. Since only the reaction time varied, all samples exhibited identical diffraction patterns, with no significant changes observed in their peak positions. It is to be noted that the four intense peaks seen are associated with the cubic-phase ITO (In_2_Sn_2_O_7−x_) substrate, aligning well with ICDD card no. 01-071-0575. Additionally, the XRD pattern of the ITO substrate is provided, along with the overlay of composite films, to verify the presence of the desired materials. It is to be noted that some minor peaks are suppressed due to the intense dominance of ITO peaks. On the other hand, the broad characteristic peaks observed at angles of 17.56 and 20.62°, having planes of (110) and (211), respectively, are associated with the existence of Prussian blue (CIF-4343748), along with some additional low-intensity peaks. This confirms the successful formation of cubic-phase Prussian blue thin-film electrodes via the chronopotentiometric technique, ensuring no trapped intermediate phases, as electrochromism involves multiple oxidation and reduction cycles [20]. The existence of broad peaks suggests nanoscale phase formation, which enhances electrochemical performance by providing a larger surface area, shorter ion diffusion pathways, and lower ion transfer resistance, reducing energy consumption for efficient switching. Moreover, characteristic peaks of PANI are additionally observed at an angle of 22.97°, corresponding to the (200) plane, along with a low intense peak at 17.4°, which is supported by the literature [21,22]. Thus, from the XRD studies, we confirmed the presence of both PB and PANI in the composite electrode.

**Figure 2:**
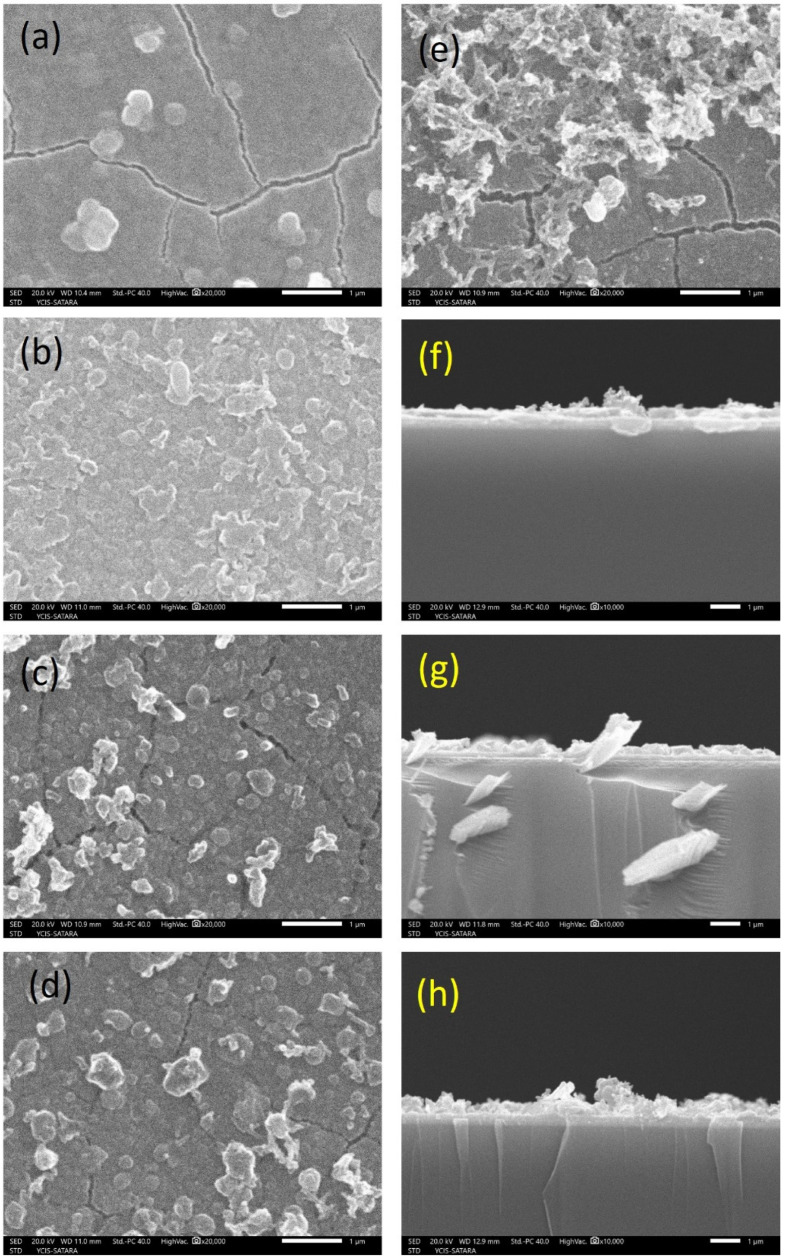
Morphological analysis of PB-PANI films synthesized at different times along with pristine Prussian blue and PANI. SEM images of (**a**) pristine Prussian blue thin-film electrode, (**b**) pristine PANI, (**c**) PB-PANI 30, (**d**) PB-PANI 60, (**e**) PB-PANI 90. Cross-sectional images of the (**f**) PB-PANI 30, (**g**) PB-PANI 60, (**h**) PB-PANI 90.

**Figure 3:**
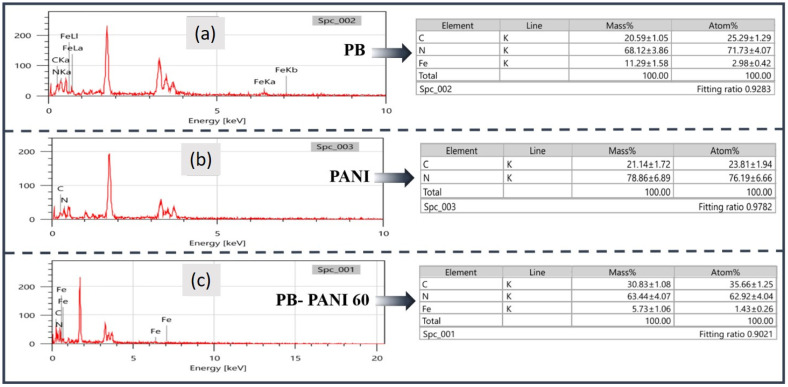
Elemental analysis of the EDA measurements of the EDS: (**a**) pure Prussian blue; (**b**) pristine PANI; and (**c**) composite PB-PANI 60.

Morphological analysis was conducted using SEM at various magnifications. Figure 2 displays SEM images at 20,000× magnification for all the samples, including bare PB and PANI (Figure 2c–e). The SEM image of a bare PB film (Figure 2a) revealed that samples exhibited compact and dense morphology composed of aggregated nanoparticles with visible nanoscale cracks on the surface. Moreover, the bare PANI film (Figure 2b) prepared at a 60 s reaction time showed a crack-free, rough, and porous structure, which might help to provide more access for electrolytes, contributing to overall electrochemical processes. Composite films such as PB-PANI30 (Figure 2c) exhibited a porous yet densely packed structure, and cracks were also visible with a very thin layer of PANI over PB, as performed with a much shorter reaction time. Conversely, sample PB-PANI60 (Figure 2d) showed a more suitable morphology for EC reactions and energy storage, with fewer cracks, a rough porous structure and lower agglomeration. Reduced agglomeration and higher access to the base layer of PB are necessary to evaluate the synergistic effect of electrochemical properties. As the reaction time increased, sample PB-PANI 90 (Figure 2e) showed the overgrowth of PANI as an agglomerated rod-like morphology on the base layer of PB with visible cracks. The topography is porous with a rod-like morphology, giving free space for ions for mobility. The cross-sectional images shown in Figure 2f–h reveal the clear layers of the PB and PANI, whereas the thickness of the PANI increases with respect to the deposition time. Furthermore, the elemental composition of all the composite films was evaluated via Energy-Dispersive Spectroscopy (EDS) analysis, confirming the presence of Fe, C, and N in the expected proportions across all samples. Additional peaks corresponding to Si, In, Sn, and O were also detected, attributed to the conductive ITO-coated glass substrate. More specifically, Figure 3a presents the EDS spectra of the PB sample with Fe, C and N elements; Figure 3b shows a PANI sample with C and N elements; and Figure 3c shows the EDS spectra of a composite film, exhibiting varying concentrations of Fe, C and N as both structures contain C and N [23]. The presence of H in the conducting polymers cannot be detected using the EDS technique. These physico-chemical analyses confirm the impurity-free formation of nanoscale PB-PANI films, making them well suited for electrochromic supercapacitor applications.

**Figure 4:**
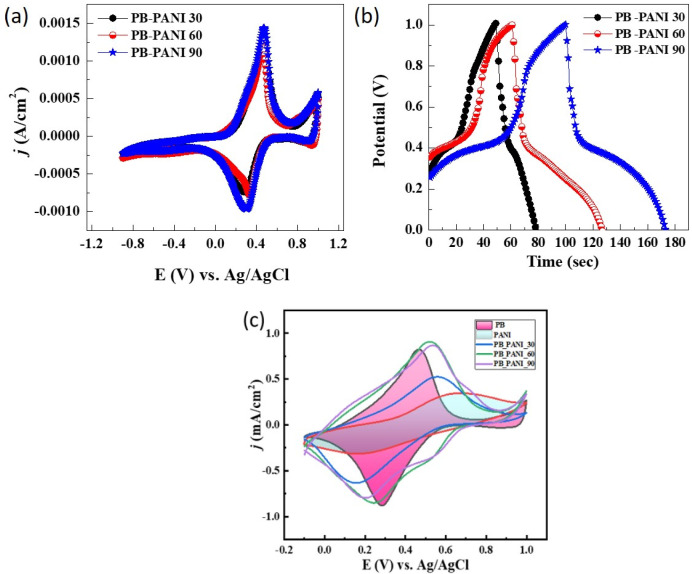
Electrochemical measurements in tree electrode system of the composite films. (**a**) Cyclic voltammetry (CV), (**b**) galvanostatic charge–discharge of PB-PANI composite thin-film electrodes fabricated at different times of 30, 60 and 90 s and (**c**) overlay of CV of PB, PANI, and PB-PANI composite thin-film electrodes fabricated at different times of 30, 60 and 90 s.

The electrochemical behaviors of the PB-PANI thin films were evaluated through the cyclic voltammetry (CV) technique. The CV measurements (Figure 4a) are performed using a three-electrode electrochemical setup in a 1 M KCl electrolyte, with the applied voltage ranging from −0.9 to 1 V at a scan rate of 25 mV/s. The KCl electrolyte was chosen primarily for the stability of the Prussian blue (PB) film, which is degrades in strongly acidic environments. In this PB–PANI composite, PB acts as the primary electrochromic center, while PANI serves largely as a conductive, ion-permeable matrix, along with a safeguarding PB layer. This matrix helps electron transport and stabilizes charge compensation during the intercalation and deintercalation of K^+^ ions. Although PANI’s intrinsic redox activity is lower in neutral KCl than in acid, it retains sufficient conductivity to support the overall composite framework. The identical voltametric profiles indicate excellent stability and repeatability of all samples. The CV curves exhibit very sharp oxidation and reduction peaks, confirming the presence of stable redox couples within the system. The oxidation peak is observed at 0.46 V, for all samples, while the reduction peak appears with a slight shift from 0.27 to 0.32 V, signifying the PB-PW redox transition. The variation in redox peak positions underlies the trapping behaviors of electrodes, which might negatively affect the EC performance. During the bleaching process, Fe^3+^ ions undergo reduction, accompanied by the intercalation of potassium ions, which plays a crucial role in the electrochromic switching mechanism [24]. The coloring followed by ion deintercalation produces an oxidation peak at 0.46 V and converts Prussian blue to Prussian green through partial oxidation. The overall process shows color switching within Prussian blue to colorless (Prussian white) for all samples. The bleached state found to be not fully transparent is due to the influence of polyaniline. However, polyaniline shows good color switching from light green to bluish green with the insertion of H^+^ ions in sulfuric acid as the electrolyte, and it is no more preferable for Prussian blue. The following formula is used to calculate the areal capacitance using cyclic voltametric data [25].

(1) $$C_{cv}=\frac{\int Idv}{2va∆V}$$

The equation above includes the applied current (*I*), scan rate (*v*), area of the active electrode (*a*), and operational potential range (Δ*V*). The obtained areal capacitance is significantly larger than previously published values for PB-based electrochromic electrodes. The sample produced with 90 sec of reaction time like PB-PANI-90 showed the highest aerial capacitance of 45.18 mF/cm^2^, surpassing PB-PANI 30 (39.47 mF/cm^2^) and PB-PANI 60 (40 mF/cm^2^).

Galvanostatic charge–discharge (GCD) tests were utilized to investigate charge storage capacity using the oxidation and reduction potentials that are acquired from CV analysis. Figure 4b illustrates an overlay of the GCD curves for all three PB-PANI electrodes recorded at a current density of 0.1 mA/cm^2^. Among all the composite films, PB-PANI90 showed a higher discharge time than PB-PANI60 and PB-PANI30. It is be noted that all samples showed two-stage phase variation in their GCD profiles. The overall trend for PB-PANI shows that electrodes with a large quantity of mass deposited have a longer involvement in the electrochemical process but lack stability. Additionally, longer reaction times diminish the film’s transparency and, as a result, its electrochromic performance. The overall CV and GCD results suggests the PB-PANI90 composites allowed sufficient growth over the base layer of PB and contributed positively to EC performance. Electrochemical performance improved with greater mass and electrolytic ions but may not be beneficial due to a lack of stability and the electrodes’ restricted transparency for electrochromic applications [26]. Figure 4c shows the overlay of cyclic voltametric curves of PB, PANI, and PB-PANI composite thin-film electrodes fabricated at different times of 30, 60 and 90 s. The curves exhibit distinct redox features, showing individual electrochemical responses. The PB electrode shows redox peaks, indicating its faradaic charge storage behavior, while the PANI electrode displays broader redox features with a more quasi-rectangular shape and a higher current response than that of PB, suggesting improved conductivity and a pseudocapacitive contribution. PB-PANI composite electrodes exhibit significantly higher current density and a larger area of CV loops, justifying a clear synergistic effect between PB and PANI. The composites show more pronounced and better-defined redox peaks, indicating improved charge transfer kinetics and a higher electroactive surface area due to effective interfacial interaction between PB nanoparticles and the PANI matrix.

**Figure 5:**
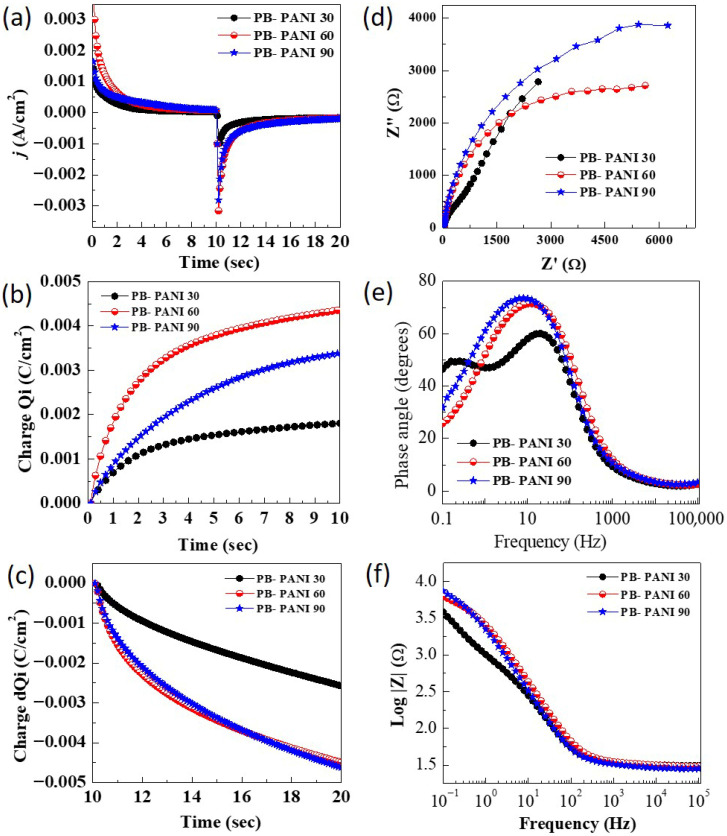
(**a**) Chronoamperometric curves, (**b**) chronocoulometric curves of the intercalation, (**c**) chronocoulometric curves of the deintercalation, (**d**) Nyquist plots, (**e**) Bode plots with phase angles, (**f**) Bode plots Z vs. frequency of all PB-PANI composites films grown at different times.

To gain deeper insight into charge kinetics and switching times, pulsed chrono-techniques such as chronoamperometry (CA) and chronocoulometry (CC) were employed in the same electrolyte bath [27]. During the chronoamperometry test (Figure 5a), a constant potential pulse was applied for a fixed duration, and the corresponding changes in current density reflecting the charging and discharging of the electrode were recorded at potential pulses of −0.4 V and 0.8 V for 10 s sequentially. A 90% drop or rise in current signifies a phase transition corresponding to the coloration or bleaching of the films. The time required for the current to change from its initial value is defined as the coloration and bleaching times. A faster response to the applied potential pulse indicates favorable conditions for real-time electrochromic applications, such as smart windows and display devices. The measured coloration/bleaching times for different PB-PANI samples were as follows: PB-PANI 30 (1.5/1.8 s), PB-PANI 60 (2.3/3.1 s), and PB-PANI 90 (0.9/1.7 s). All films exhibited optimal reaction times, demonstrating their potential suitability for electrochromic applications. The switching time affects the coloration efficiency values as it shows which charges are sufficient for color switching. The lower the charges, the higher the value of coloration efficiency. Additionally, chronocoulometric tests (Figure 5b,c) were utilized during charge intercalation at an applied potential of 0.8 V to quantify the charges inserted and extracted during electrochemical redox reactions. These measurements are crucial for evaluating key parameters such as coloration efficiency, which reflects the material’s capability to undergo coloration and discoloration during ion insertion and extraction in oxidation and reduction processes. The special advantage of such polymeric materials is that the materials exhibit color switching upon the insertion of a much smaller number of charges, theoretically increasing the efficiency of this class of materials [28].

Electrochemical impedance spectroscopy (EIS) was conducted to analyze the electronic transport properties along with its effect on the electrochemical performance of the electrodes and gain insights into charge storage dynamics during charge transfer processes over a range of applied frequencies. This is a non-destructive technique that helps to justify improved performances because of the ease of charge transport. The EIS measurements were conducted within a frequency range of 100 kHz to 0.1 Hz, with an AC amplitude of 10 mV. Nyquist plots for the composites PB-PANI are depicted in Figure 5d, and the extracted EIS parameters, including equivalent series resistance/solution resistance (*R_s_*) and phase angle (ϕ), were summarized in Table 1. All prepared samples showed impedance of less than 35 Ω, indicating good charge transfer behaviors. The *R_s_* values are found to be 31.07 Ω (PB-PANI 30), 30.45 Ω (PB-PANI 60) and 28.21 Ω (PB-PANI 90). The film prepared at 90 sec with higher variations showed less resistance to charge transport. Although this is not a significant difference but justifies the higher energy storage ability of the PB-PANI 90 sample. The composites prepared with PANI did not show visible semicircles. Thus, the absence of a semicircle suggests low charge transfer resistance at the electrode–electrolyte interface due to fast interfacial kinetics and good electronic/ionic transport within the composite films. Rather, a nearly linear response in the low-frequency range dominates the spectra, which is typical of capacitive or diffusion-controlled behavior, often seen in electrochromic electrodes based on conducting polymers and Prussian blue. Therefore it is difficult to calculate charge transfer resistance. The sample with a lower reaction time exhibited higher resistance, hindering EC performance. Moreover, the samples did not show a straight line at lower frequencies, indicating the dominance of the surface charge storage processes. Bode plots were analyzed to understand charge storage behaviors from the phase angle values shown in Figure 5e. The obtained values of the phase angle for composites PB-PANI 30, PB-PANI 60 and PB-PANI 90 are −73.49°, −71.41° and −60.01°, respectively. The value of the phase angle displays processes involved in the charge storage as 0° for resistive, −90° for capacitive and −45° for the diffusion type of charge storage [29,30]. The optimized samples, PB-PANI 90, have a value of −60.01°, describing mixed battery-type charge storage, justifying the distinct indication of redox peaks. The frequency-dependent impedance Bode plot (Figure 5f) analysis shows lower dependence on impedance for the PB-PANI 30 sample but lagging energy storage among the PANI composites. The SEM images reveal that increasing the PANI content progressively modifies the microstructure of the composite, leading to changes in surface roughness, particle connectivity, and the effective interfacial area between PB and the PANI matrix. These morphological features directly influence the electrochemical impedance characteristics. Specifically, the absence (or significant suppression) of a high-frequency semicircle in the Nyquist plots shows low charge transfer resistance (Rct), which can be attributed to the close interfacial contact between PB particles and the conductive PANI network, facilitating rapid electron transport across the interface.

In the low-frequency region, the near-linear or inclined impedance response suggests that electrochemical behavior is dominated by a combination of capacitive charge storage and ion-diffusion-controlled processes. The PANI matrix contributes primarily to capacitive behavior through fast surface redox reactions and electronic conduction, whereas PB contributes diffusion-controlled charge storage associated with K^+^ intercalation/deintercalation within its lattice. Variations in slope and impedance magnitude among PB–PANI 30, 60, and 90 reflect differences in ion transport pathways and diffusion lengths arising from compositional and morphological changes. At higher PANI content, the presence of PANI domains enhances charge storage capacity but may introduce longer diffusion paths for ions, whereas intermediate compositions provide a more balanced microstructure with effective ion access and electronic percolation. These structure-dependent impedance features are consistent with the observed CV and GCD trends and support a synergistic charge storage mechanism within the composite.

**Figure 6:**
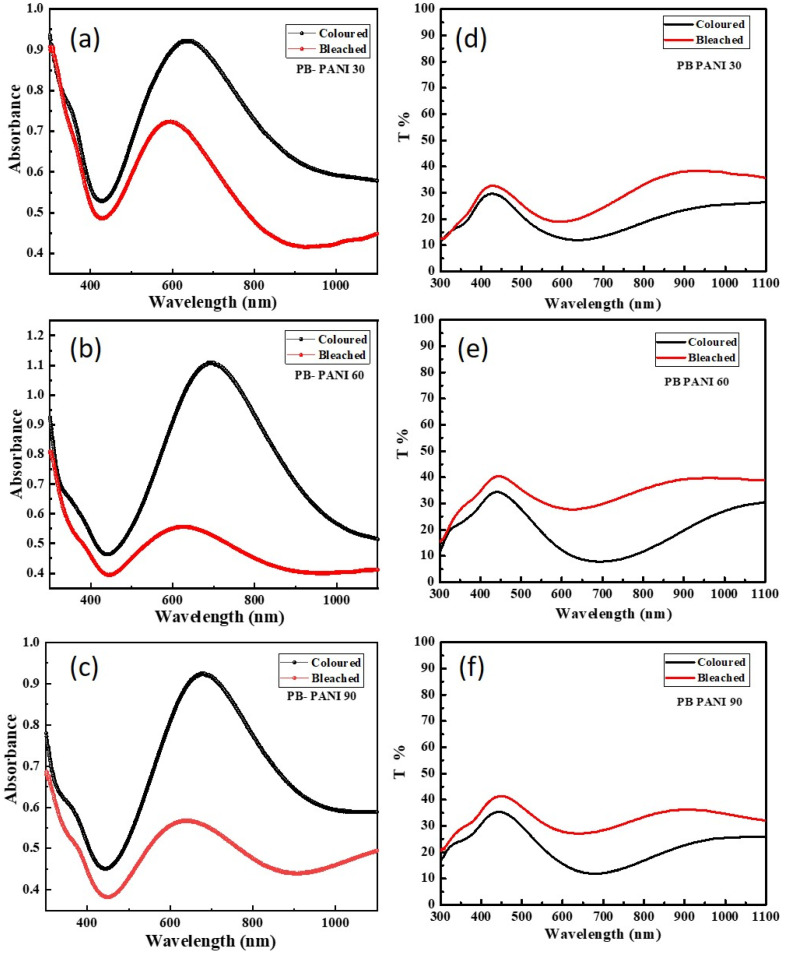
Ex situ transmittance and absorbance spectra of (**a**,**d**) PB-PANI 30, (**b**,**e**) PB-PANI 60, and (**c**,**f**) PB-PANI 90 in the colored and bleached states to estimate coloration efficiency, optical density and optical modulations.

To study the electrochromic effect, optical studies, namely absorption and transmittance of the prepared films in colored and bleached states, were conducted using a UV–Visible spectrophotometer. The electrodes alternated between colored and bleached states by applying a potential pulses of 1V and −0.5V, respectively, using the chronoamperometric technique. Figure 6a–c show the absorbance spectra of all the composite electrodes, which providing insights into the electronic transitions and charge transfer processes occurring within the film. The well-defined absorption bands correspond to the charge transfer of PB and the PANI. Among the composites, the film synthesized at 60 s deposition time exhibits the highest absorbance (ΔA) between bleached and colored states, correlating well with its maximum optical contrast observed in the transmittance spectra. In contrast, the 30 s film shows lower absorbance due to insufficient film thickness and incomplete coverage, while the 90 s film, despite higher overall absorbance, shows relatively reduced optical modulation, likely due to increased scattering losses and limited ion diffusion in thicker films. Following this process, ex situ transmittance measurements (Figure 6d–f) were conducted over a wavelength range of 300–1100 nm. The results showed that during reduction, the electrodes transitioned to a bleached state (Prussian white) due to K^+^ ion intercalation and electron transfer, facilitating the Fe^3+^ to Fe^2+^ transition [31,32,33]. Conversely, when a positive potential was applied, the films regained their characteristic blue color (Prussian blue) due to ion and electron deintercalation. The composite samples show lower transmittance than the reduced state of bare PB, which is the combined effect of the composite. The variation in transmittance between the colored and bleached states plays a crucial role in assessing electrochromic performance. Specifically, at 630 nm, the transmittance in the colored state underscores the suitability of these electrodes for electrochromic display applications. Among the polyaniline composite samples, PB-PANI 90 exhibits the best performance, achieving balanced transmittance modulation, alongside high coloration efficiency. By analyzing the transmittance data, essential electrochromic parameters, including contrast ratio (*CR*), optical density difference (Δ*OD*), and coloration efficiency (*CE*), are calculated using the following equations [34,35].

(2) $$CR=\frac{T_{b}}{T_{c}}$$

(3) ∆OD=log TbTc λ=630nm

(4) $$CE=\frac{∆OD}{∆Q}$$

(5) $$∆Q=\int_{t1}^{t2}I\left(t\right).dt$$

**Figure 7:**
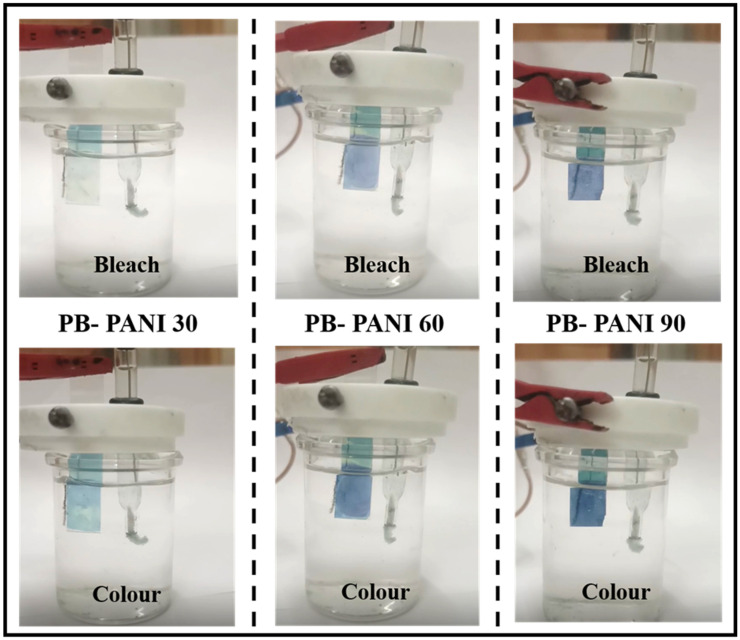
Digital photos of coloration and bleaching states of composite films of PB-PANI 30, PB-PANI 60, and PB-PANI 90, respectively.

The optical response of the samples is characterized by their transmittance at 700 nm, where *T_b_* represents the bleached state and *T_c_* corresponds to the colored state, while *Q* denotes the intercalated charges. Among all the samples, PB-PANI 60 exhibited the highest optical modulation of 19.4%, demonstrating a pronounced electrochromic effect. The PB-PANI 90 exhibited the highest coloration efficiency of 461.39 cm^2^/C, which is significantly higher than the other prepared composites as well as previously reported materials. In comparison, PB-PANI 30 and PB-PANI 60 showed optical modulations of 8.7% and 14.7% with *CE* values of 281.38 cm^2^/C and 199.17 cm^2^/C, respectively. Figure 7 shows digital photo images of colored and bleached states of PB-PANI 30, PB-PANI 60, and PB-PANI 90. The results indicate that increasing the reaction time leads to greater optical modulation as more material is deposited over time, forming a thicker layer. A thicker film allows for a more intense coloration in the colored state, enhancing the contrast between the bleached and deep-blue states. The results of optical studies are shown in Table 2. However, the sample with higher reaction time (90 s) has a higher *CE* value but shows less areal capacitance, indicating the imbalance between dual functionalities. Coloration efficiency denotes the change in optical density per unit of injected charge, whereas optical modulation depends on the absolute change in transmittance across the film. In the case of PB-PANI 90, the higher *CE* indicates that the injected charge is utilized more effectively to induce optical density changes, which can be attributed to the increased fraction of electroactive PANI domains and efficient interfacial charge transfer within the composite. However, the higher PANI loading also results in a thicker and more optically dense film, leading to increased baseline absorption and scattering losses. As a result, although PB-PANI 90 exhibits strong charge-induced optical contrast on a per-charge basis, the absolute transmittance change is partially limited by reduced light penetration. In contrast, PB-PANI 60 forms a comparatively thinner and more homogeneous layer, allowing more effective light transmission and, thus, slightly higher optical modulation despite its lower *CE*. Additionally, the effective electroactive area contributing to optical modulation may differ from the total charge-storing area, particularly in thicker films, where ion-accessible regions near the surface dominate the optical response. With optimized electrochromic effects, energy storage ability is the main aspect as it allows for dual functionality as well as self-charging devices feasibly.

**Figure 8:**
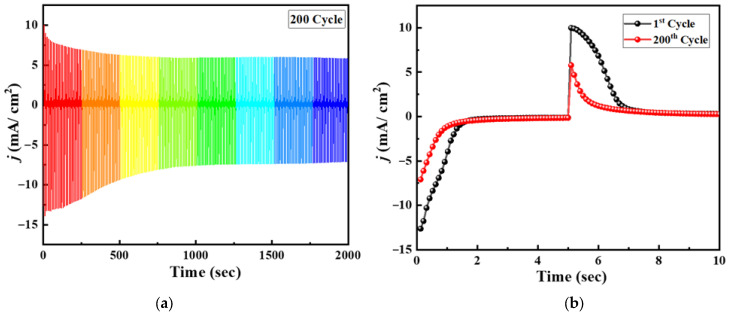
(**a**) Electrochromic stability of the composite electrode for 2000 consecutive CA cycles with 2000 s of working time. (**b**) Comparative overlay of CA of 1st and 200th cycle.

The electrochemical and electrochromic stability of the optimized PB-PANI composite electrode fabricated with a deposition time of 90 s was evaluated to assess its suitability for long-term operation. Figure 8a shows the electrochromic stability of the composite electrode over 2000 consecutive chronoamperometric cycles with 2000 s of working time, and Figure 8b depicts the comparative overlay of CA for the 1st and 200th cycle. These stability plots reveal that the composite maintains a highly consistent response over repeated cycling, indicating good structural integrity and reversible electrochemical behavior. The film stabilizes during the first 50 cycles, showing only a marginal decrease in current response after that, suggesting minimal degradation of electroactive sites. The retention of the overall curve shape throughout the cycles confirms that the redox reactions associated with both PB and PANI remain largely reversible, with no significant distortion or peak shifting observed. This stability can be attributed to the strong interfacial interaction between PB and the PANI matrix, which effectively buffers structural changes occurring during repeated ion insertion and extraction. Overall, our study concluded with an improvement in both energy storage and the electrochromic effects of Prussian blue films via successful composition with polyaniline. The other color transitions of Prussian blue such as green and brown are intentionally avoided by shortening the potential window due to instability in the electrolyte. This study suggests PB-PANI composites show greater energy storage performances as well as improved electrochromic results.

## 4. Conclusions

In this study, the effect of the electrodeposition time of polyaniline on the electrochromic performance of Prussian blue was systematically evaluated. The Prussian blue–polyaniline composites were successfully deposited on ITO substrates via chronopotentiometry followed by chronoamperometry techniques. The formation of composites is confirmed by X-ray diffraction (XRD). The SEM images show the morphological modifications caused due to composition with polyaniline. Among polyaniline composites, the PB-PANI 90 sample exhibited the highest coloration efficiency, up to 461.39 cm^2^/C, and higher areal capacitance of 45.18 mF/cm^2^ with optical modulation of 19.4%. All samples demonstrated rapid switching times of less than 3 s. The proper optimization of conducting polymers such as polyaniline over Prussian blue can achieve a good balance between composites, along with morphological tuning for advanced dual-functional devices with next-generation smart systems.

## Figures and Tables

**Table 1:** Supercapacitive and impedance analysis of PB-PANI composite film electrodes.

Sample Name	Sc (mF/cm^2^) by CV	*R_s_* (Ω)	ϕ (Degree)
PB-PANI 30	39.47	31.07	73.49
PB-PANI 60	39.95	30.45	71.41
PB-PANI 90	45.18	28.21	60.01

**Table 2:** Electrochemical parameters such as switching times, contrast ratio, optical modulation and coloration efficiency of all composites of PB-PANI electrodes.

Sample Name	*T_c_*/*T_b_* Sec	*CR* at 700 nm	ΔT % at 700 nm	*CE* (log) cm^2^/C
PB-PANI 30	1.5/1.8	1.820	8.7	281.38
PB-PANI 60	2.3/3.1	3.820	19.4	199.17
PB-PANI 90	0.9/1.7	2.347	14.7	461.39

## Data Availability

The original contributions presented in this study are included in the article. Further inquiries can be directed to the corresponding authors.
